# Does Preventing Obesity Lead to Reduced Health-Care Costs?

**DOI:** 10.1371/journal.pmed.0050037

**Published:** 2008-02-05

**Authors:** Klim McPherson

## Abstract

The author discusses the implications of a new study that used data from The Netherlands to simulate the annual and lifetime medical costs attributable to obesity.

In a study in this issue of *PLoS Medicine*, Pieter van Baal and colleagues used data from The Netherlands to simulate the annual and lifetime medical costs attributable to obesity [1]. They also compared these costs to those attributable to smoking as well as to the medical costs associated with healthy, living persons (defined as non-smokers with a body mass index in the range of 18.5 to less than 25 kg/m^2^). The researchers explored the question of whether reducing obesity would lead to reduced or increased health-care costs

## The Main Findings

The study found that although annual health-care costs are highest for obese people earlier in life (until age 56 years), and are highest for smokers at older ages, the ultimate lifetime costs are highest for the healthy (nonsmoking, nonobese) people. Hence the authors argue that medical costs will not be saved by preventing obesity.

Their results tell us that that life expectancy from age 20 is reduced by 5 years for obese people and 7 for smokers. The consequence is that healthy people live to incur greater medical expenditure subsequently on average, more than compensating for the earlier excess expenditure related to obesity or smoking.

The recent Foresight report on obesity in the United Kingdom looks ahead to 2050 and stresses that obesity rates are rising, that the determinants of these rises are complex and go much further than individual control of diet and exercise, and that if nothing changes costs to the health service and society will increase as a direct consequence [2]. Yet van Baal and colleagues' study suggests that obese people cost *less* to health services than nonobese people. Smokers cost still less. Clearly at a time when obesity is deemed a major health issue, in part because of its putative health-care costs, the results of this new study sound paradoxical.

**Linked Research Article**

This Perspective discusses the following new study published in *PLoS Medicine:*

van Baal PHM, Polder JJ, de Wit GA, Hoogenveen RT, Feenstra TL, et al. (2008) Lifetime medical costs of obesity: Prevention no cure for increasing health expenditure. PLoS Med 5(2): e29. doi:10.1371/journal.pmed.0050029

Using a simulation model, Pieter van Baal and colleagues conclude that obesity prevention leads to a decrease in costs of obesity-related diseases, but this is offset by cost increases due to diseases unrelated to obesity in life-years gained.

## How Should We Interpret These Results?

In a sense, Van Baal and colleagues' study is a useful antidote to current concerns. But let us be clear: it does not attenuate them. Obese people cost less because individuals die younger and hence with less chronic morbidity associated with old age. This is a useful thing to know, but how might it affect public health strategies for obesity? In particular, does it mean that concerns about increasing population obesity are misplaced, as least as far as health-service costs are concerned?

Sadly not. Examine an obese population and a lean population of the *same* age and sex distribution, and the former will incur far greater health-care costs throughout the life course. Much more diabetes, and more cardiovascular disease and cancer will occur amongst the obese—even amongst the older obese [3]. Compare health-care costs now with those thirty years ago, and—holding everything but obesity constant—the current population costs much more to the health sector than it did then [4]. Moreover, quite apart from health-care costs, the other costs to society from obesity are also greater because of absences from work due to illness and employment difficulties; these costs amount to considerably more than health-care costs [5]. It is not clear that these extra costs are intrinsically related to health-care costs, but they are currently estimated to be around four times as great in obese than in lean people [5].

But is it worth knowing that obese individuals are cheaper than lean ones for the health sector in the *long run*? What does that mean for clinical or public health practice? Unless taxpayers take their cue and eat too much and exercise too little in order to reduce their tax burden, it has no particular implications. And the study may reinforce the cynical view that governments may be reluctant to intervene when doing so implies greater health expenditure on each individual through their life. We have to be clear, therefore, about the distinction between lower lifetime health costs associated with obese individuals and higher costs of obese populations.

## Quality-Adjusted Life Years

Most people want to live a longer life, and they do not consider the consequences to society. But a major goal of life is to maximise one's total quality-adjusted life years (QALYs). Unfortunately, this new study provides little insight into the total QALYs associated with obesity and smoking. Certainly those who are obese and those who smoke will live fewer years on average, but will these people be compensated by enriched quality of their fewer years? Available evidence suggests strongly that quality of life, quite apart from increased illness rates, is considerably compromised by obesity [6].

We know already that health expenditure is high amongst the elderly, especially so amongst those who die of old age [7]. Van Baal and colleagues' study confirms the high medical costs of living to old age, but the results should not be interpreted as justifying the “cost savings” to society of dying younger. And, as the authors point out, it would be wrong to interpret the findings as meaning that public-health prevention (e.g., to prevent obesity) has no benefits. As the authors say, whilst prevention may not be “a cure for increasing expenditures—instead it may well be a cost-effective cure for much morbidity and mortality and, importantly, contribute to the health of nations” [1].

The balance of QALYs gained or lost, although not addressed in the van Baal study, could have been addressed—at least approximately. The subsequent analyses ought to tell us what this balance looks like for successful projects to contain the growth of obesity in populations. We need to know this balance both for lifetime gains and losses in quality of life and for comparisons of aggregated quality of life between more-obese societies and less-obese ones. Unlike the health expenditure comparison considered by van Baal and colleagues, the signs are that the overall balance will be entirely positive for obesity prevention—for individuals and populations.

## Conclusion

Much research is still needed, in the United Kingdom at least, on the costs of being overweight (BMI >25) as opposed to being obese (BMI >30) for both the health sector and society as a whole. But van Baal and colleagues have enhanced our understanding of these issues. Translating individual costs and benefits to societal costs and benefits is never straightforward, and their study successfully emphasises the problem. People tend to have views about health that relate to their individual experiences, including personal lifetime expenditures. This new study serves to remind us that merely multiplying such estimates by population sizes of several million does not illuminate real public-health dilemmas. And this kind of simplification leaves out the numerous societal implications of obesity, which themselves are massively complex [9]. Governments need to understand that public-health policies affect more than merely the sum of individual health, and sadly require greater courage to implement than does treating the sick.

## Abbreviations

QALY: quality-adjusted life year
